# Treatment With Probiotic Bacteria Does Not Diminish the Impact of a *Cystoisospora suis* Challenge in Suckling Piglets

**DOI:** 10.3389/fvets.2018.00313

**Published:** 2018-12-12

**Authors:** Christine Unterweger, Lukas Schwarz, Miriam Viehmann, Alexandra von Altrock, Gerhard F. Gerlach, Karl-Heinz Waldmann, Anja Joachim, Isabel Hennig-Pauka

**Affiliations:** ^1^Department for Farm Animals and Veterinary Public Health, University Clinic for Swine, University of Veterinary Medicine, Vienna, Austria; ^2^Clinic for Swine, Small Ruminants, Forensic Medicine and Ambulatory Service, University of Veterinary Medicine Hannover, Hanover, Germany; ^3^Innovative Veterinary Diagnostic GmbH, Hannover, Germany; ^4^Department for Pathobiology, Institute of Parasitology, University of Veterinary Medicine, Vienna, Austria; ^5^Field Station for Epidemiology, University of Veterinary Medicine Hannover, Bakum, Germany

**Keywords:** *Cystoisospora suis*, *Clostridium perfringens* type A, probiotics, suckling piglets, gut stabilization

## Abstract

Colonization of newborn piglets with beneficial and ubiquitous microorganisms in combination with colostral passive immunity is the prerequisite for development of immunity and gut maturation. In this study living strains of *Clostridium perfringens* type A (*Cp*A) and non-pathogenic *Escherichia* (*E*.) *coli* strains harvested from healthy piglets were administered to piglets prior to first colostrum intake in order to prevent disease caused by pathogenic variants of the same bacterial species by competitive exclusion. In addition, it was investigated whether these potential beneficial colonizers were able to prevent harmful effects of infection with *Cystoisospora (C.) suis* as a primary invasive pathogen. In a first trial, half of the piglets from four litters were treated with a bacterial cocktail consisting of two *E. coli* and four *Cp*A strains immediately after birth on two consecutive days, while the other half of the litters served as control group. In a second trial, piglets were treated following the protocol of the first trial, and additionally all piglets were infected 4 h after the end of littering with ~1,000 sporulated oocysts of a *C. suis* laboratory strain. General health, body weight development, fecal consistency and, in the second trial, oocyst excretion were monitored from birth until weaning. No adverse effects of the cocktail on the health status were observed. Treated piglets of the first trial showed a higher average daily weight gain until weaning. In the second trial, no significant differences were found with respect to average daily weight gain, fecal consistency, amount, and duration of oocyst excretion assessed in daily samples. In treatment group 51.1% and in the control group 38.5% of the fecal samples were positive for oocysts in autofluorescence. The average duration of oocyst excretion was longer in treatment group (7.7 days) than in control group (5.6 days). Application of bacterial cocktail could not effectively minimize disease symptoms caused by *C. suis*. There was a trend toward an increase in severity of disease symptoms in treated pigs, suggesting that the synergism between *Cp*A and *C. suis* was independent of the bacterial strains, but is exclusively dominated by the pathogenic effect of *C. suis*.

## Introduction

During birth, the sterile digestive tract of piglets is colonized by bacteria from the vaginal fluids (1). Culture-based studies on the development of the intestinal microbiota in suckling piglets did not accurately reflect microbial diversity, because ~60% of microbiota are considered as not cultivable (2). Nevertheless it could be shown that the gut flora of piglets in the first days of life is rich in *Enterococcus* spp., *Escherichia coli*, and *Clostridium perfringens*, early colonizers of the gut that are replaced to a large extend by other bacterial species until the end of the suckling period (3, 4). In recent years novel molecular techniques have been developed to study intestinal microbial ecology and shifts in microbial communities over time (4). Already upon weaning site-specific colonization of the gut has led to differentiation of luminal and mucosa-attached microbiota in ileum, caecum and colon, which is dominated by Bacterioidetes, Firmicutes, and Proteobacteria (5). Specifically, Prevotellaceae (Bacteroidia) lead to the exclusion of Enterobacteriaceae which is decisive for intestinal health (5). In addition, Clostridia (members of the phylum Firmicutes) are highly effective producers of short chain fatty acids which decrease the pH and reduce the growth of Enterobacteriaceae (5). Gut maturation and establishment of a beneficial microbiota is influenced by exposure to bacteria from the environment as well as the mother during suckling, by individual factors and by environmental parameters, e.g., temperature (6, 7). In parallel to the colonization with harmless, beneficial, and ubiquitous microorganisms, piglets also ingest pathogenic and facultative pathogenic microorganisms early in their life. Diarrhea caused by virulent (enterotoxigenic or enteropathogenic) *E. coli* strains, as well as by *C. perfringens* Type A (*Cp*A) are common problems in the first weeks of life of piglets (8, 9), especially in large litters (10).

In contrast to *E. coli* and *Cp*A, *Cystoisospora suis* (syn. *Isospora suis*) is considered to be a primary and obligate pathogen of the gastrointestinal tract of suckling piglets. *Cystoisospora suis* is an early colonizer and known as the causative agent of neonatal porcine cystoisosporosis (coccidiosis). Main histopathological findings during disease are atrophy and necrosis of the villi in the small intestine. Clinically, piglets frequently show yellowish to grayish pasty-to-watery non-hemorrhagic diarrhea, dehydration, depressed weight gain or even weight loss, and wasting (11). First signs of disease usually occur between day 7 and 11 of life (11). In suckling piglets a high morbidity, but a moderate mortality within a litter is considered to be typical (12). *Cystoisospora suis* is also an important factor for the development of multifactorial diarrheic disease or wasting in suckling piglets, usually in combination with virulent *E. coli* strains, *C. perfringens* types A and C or different viruses (13–15). It is assumed that *C. suis* as a primary pathogen is responsible for pre-injury of the gut mucosa, which facilitates adhesion of other pathogens. The gut mucosal barrier also becomes permeable for other potentially hazardous pathogens after primary alterations caused by *C. suis* (16). In a recent Japanese case report a negative synergistic effect of *C. suis* and virulent *E. coli* strains in coinfection was hypothesized, because suckling piglets developed severe diarrhea (14). Pathomechanisms of interaction between the two pathogens have not been elucidated so far.

In chicken, the influence of coccidiosis on the diversity of gut microbiota in general is well documented (17–19). Several studies in chicken revealed a correlation between the incidence of necrotic enteritis and a co-infection with clostridia and coccidia (20–23). Also previous studies in pigs clearly showed a correlation between infection with *Cp*A and severity of disease during cystoisosporosis (15, 24, 25). Simultaneous infection with *C. suis* and *Cp*A soon after birth resulted in severe clinical signs and increased mortality, which was presumed to be the consequence of interactions between the two pathogens, as alterations due to *C. suis* infection may improve growth conditions for *C. perfringens* (22). Up to now this hypothesis is supported only by empirical field studies (25).

Until now, only a limited number of studies in pigs have demonstrated positive effects of potential probiotic bacteria administered during the first days of life in order to shape a beneficial microflora (26, 27). In the present study, non-pathogenic *E. coli*- and *Cp*A*-*strains isolated from healthy pigs were given orally to suckling piglets prior to first colostrum intake. The hypothesis was that beneficial effects of *E. coli* and *Cp*A would lead to stabilization of gut microflora by competitive exclusion of their pathogenic counterparts. In addition, it was hypothesized that the administered non-pathogenic *Cp*A and *E. coli* strains are not increasing disease severity after experimental infection with *C. suis*, but lead to a reduction of clinical signs due to their beneficial effect on the development of gut microbiota and gut immunity.

## Materials and Methods

### Ethics Statement

The first part of the study, in which the beneficial effect of the bacterial cocktail was evaluated, was approved by the Lower Saxony State Office for Consumer Protection and Food safety (Certificate: 41.3-63003–01/2013), while the *C. suis* challenge experiments were approved by the institutional ethics committee and the Advisory Committee for Animal Experiments (§12 of Law for Animal Experiments, Tierversuchsgesetz–TVG) of the University of Veterinary Medicine Vienna and the Austrian Federal Ministry for Science and Research (reference number BMWF-68.205/0185-II/3b/2012).

### Bacterial Cocktail Composition

A bacterial cocktail hypothesized to have beneficial effects on the gut microflora of suckling piglets due to competitive exclusion of respective virulent strains was produced as described elsewhere (28). Bacterial strains had been harvested from healthy piglets (Table 1). Four *E. coli* strains without known genes for toxins or fimbriae and two different *Cp*A strains were included. Both *Cp*A strains positive for β2-toxin genes showed no toxin production *in vitro* (28).

**Table 1:** Components of a single dose of probiotic bacterial cocktail, stored as a lyophilisate, mixed with 154 mM NaCl about 30 min before oral application (29).

**Strain no**.	**Composition**	**Colony-forming units/ml**
1	*C- perfringens* type A, positive for α–and β 2-toxin genes—no toxin production *in vitro*	10^6^
2	*C- perfringens* type A, positive for α–and β 2-toxin genes—no toxin production *in vitro*	10^6^
3	*E. coli* A, no genes for toxins or fimbriae	10^6^
4	*E. coli* B, no genes for toxins or fimbriae	10^6^
5	*E. coli* C, no genes for toxins or fimbriae	10^6^
6	*E. coli* C, no genes for toxins or fimbriae	10^6^

### Animals and Experimental Design

Sows used in this study originated from different commercial farms with unknown status of pathogen exposure. All sows were in good body condition and health status. One day before farrowing, sows were fixed in farrowing crates, had free access to water and were fed with commercial feed for lactating sows free of antimicrobials and other pharmaceutical substances. Sows and their litters used in the two consecutive trials were continuously observed around farrowing. Piglets received colostrum and milk from their mothers, had free access to water and were additionally fed with piglet starter from the 2nd week of life. On day three of life all piglets received 200 mg of iron dextran subcutaneously. The day of birth was defined as study day (SD) 1. All piglets were clinically checked for overall health, individually marked with ear tags for identification and weighed. Healthy piglets weighing at least 0.9 kg at birth were included in the study. Immediately after birth and before first colostrum intake, every second individual piglet was orally treated with 1.5 ml of the bacterial cocktail suspension (groups A), while every other second piglet received 1.5 ml sterile 154 mM sodium chloride (groups B). Treatment with the bacterial cocktail was repeated on SD 2 and SD 3.

#### Trial 1

Four pregnant sows were housed in the animal husbandry facilities of the University of Veterinary Medicine, Hannover, Foundation and were observed around farrowing until weaning with 29–35 days (end of the study). Piglets (Group A1 = 16 piglets, group B1 = 17 piglets) were weighed on the day of birth and again on SD 7, 14, 21, and at weaning. Clinical observations including fecal consistency upon defecation were recorded every day until the termination of the study (see Table 2 for details).

**Table 2:** Evaluated parameters.

**Parameter**	**Trial 1**	**Trial 2**
Total number of animals	33	18
Treated (A)	16	9
Untreated (B)	17	9
General health inspection	SD 1 until weaning = end of study (SD 29-34)	SD 1-22 (end of study)
Body weights	SD 7, 14, 21, weaning	SD 1, 8, 15, 22
Fecal consistency (scoring: see text)	Daily upon inspection	Daily individual samples SD 5-21
Oocyst excretion—qualitative	n/a	Daily individual samples SD 5-21, autofluorescence detection
Oocyst excretion—quantitative	n/a	Daily individual samples SD 5-21, modified McMaster technique

*n/a, not applicable*.

#### Trial 2

Two pregnant crossbreed gilts were housed in the large animal facilities at the Institute of Parasitology of the University of Veterinary Medicine Vienna, Austria. Immediately after birth and before first colostrum intake, animals were weighed, randomized and treated as described above (group A2: *n* = 9, group B2: *n* = 9). Within 4 h after the end of the individual sow's littering all piglets were orally infected with a 2 ml suspension containing 1,000 sporulated oocysts of a *C. suis* laboratory strain (Wien I) maintained routinely at the Institute of Parasitology, University of Veterinary Medicine Vienna, Austria (30). Piglets were weighed at birth and again on SD 8, SD 15, and SD 22. Clinical observations were recorded every day until the termination of the study on SD 22 (see Table 2).

From SD 5 to SD 21, fecal samples were taken individually and judged immediately using a fecal score system. Fecal consistency was graded as firm (fecal score 1, FS 1), pasty (FS 2), semi-liquid (FS 3), or liquid (FS 4) with FS 3 and 4 considered as diarrhea (24).

Oocyst excretion was identified by autofluorescence microscopy (31, 29). Each sample was examined completely or until oocysts were detected. Positive samples were examined by a modified Mc Master technique (23, 29) to determine the oocysts per gram of feces (OpG). For details on the parameters see Table 2.

### Statistical Analyses

Statistical analyses were done using PASW statistics ver. 24 (SPSS Inc., Chicago, USA). Data were split to sampling days and analyzed with a Mann-Whitney two-sample test comparing cocktail-infected piglets (group A2) and sham-treated control piglets (group B2). Group influence was tested in addition by one-way analysis of variance (ANOVA). Significance was assumed for *p* < 0.05.

## Results

### Trial 1

**Figure 1:**
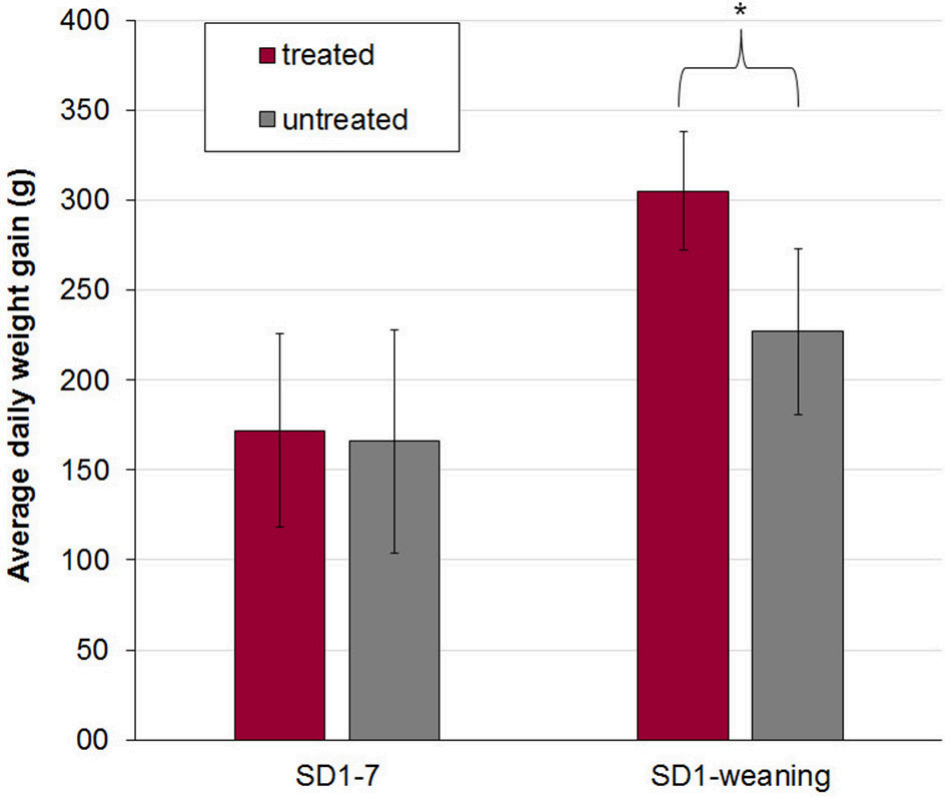
Mean and standard deviation of average daily weight gain in treated (*n* = 16) and untreated (*n* = 17) piglets out of four litters from birth to day 7 (SD 1–7, litter 1–4) and from birth to weaning (SD 1-weaning, litter 1 and 2). The bacterial cocktail was applied orally to half of the piglets of each litter immediately after birth and on day two and three of life. The other half of the piglets served as a control group and got 154 mM NaCl. Asterisk indicates a significant difference between treated and untreated piglets. In litters 3 and 4 all piglets had to be treated after day 10 of life due to respiratory disease or arthritis and were excluded from further evaluation of body weight development.

Treated and untreated piglets from litters 1 and 2 were in a good health status until weaning. The vitality of piglets of litters of sow 3 and 4 was slightly reduced after birth. Four piglets were born with splay legs and had to be assisted in colostrum uptake in the first hours of life. No adverse effects after application of the bacterial cocktail treatment and no diarrhea were observed in piglets during the suckling period. On SD 1–2 single piglets showed pasty feces (FS 2) but no diarrhea (mean FS ± standard deviation: day 1 1.15 + 0.34, day 2: 1.06 + 0.24). After that, FS = 1 in all piglets with no group differences. Antibiotic treatment of arthritis and slight respiratory disorders was necessary after SD 10 in litters 3 and 4, so that piglets had to be excluded from further evaluation. Average daily weight gains of piglets in litters 3 and 4 were only evaluated from day of birth until day 7 of life and were not different between treated and untreated animals (Figure 1). A significant effect of the sow on piglets' body weight at birth (*P* < 0.0001), on days 2 (*P* < 0.0001) and 3 (*P* = 0.005) of life as well as on average daily weight gain until day 7 (*P* = 0.029) was determined by one-way analysis of variance (ANOVA). For piglets from litters 1 and 2 no difference in birth weight between treated and untreated pigs was determined, while the difference in average daily weight gain from birth until weaning was significantly higher in treated pigs (*P* < 0.017) (Figure 1).

### Trial 2

#### Clinical Observations, Body Weights and Fecal Examination

**Figure 2:**
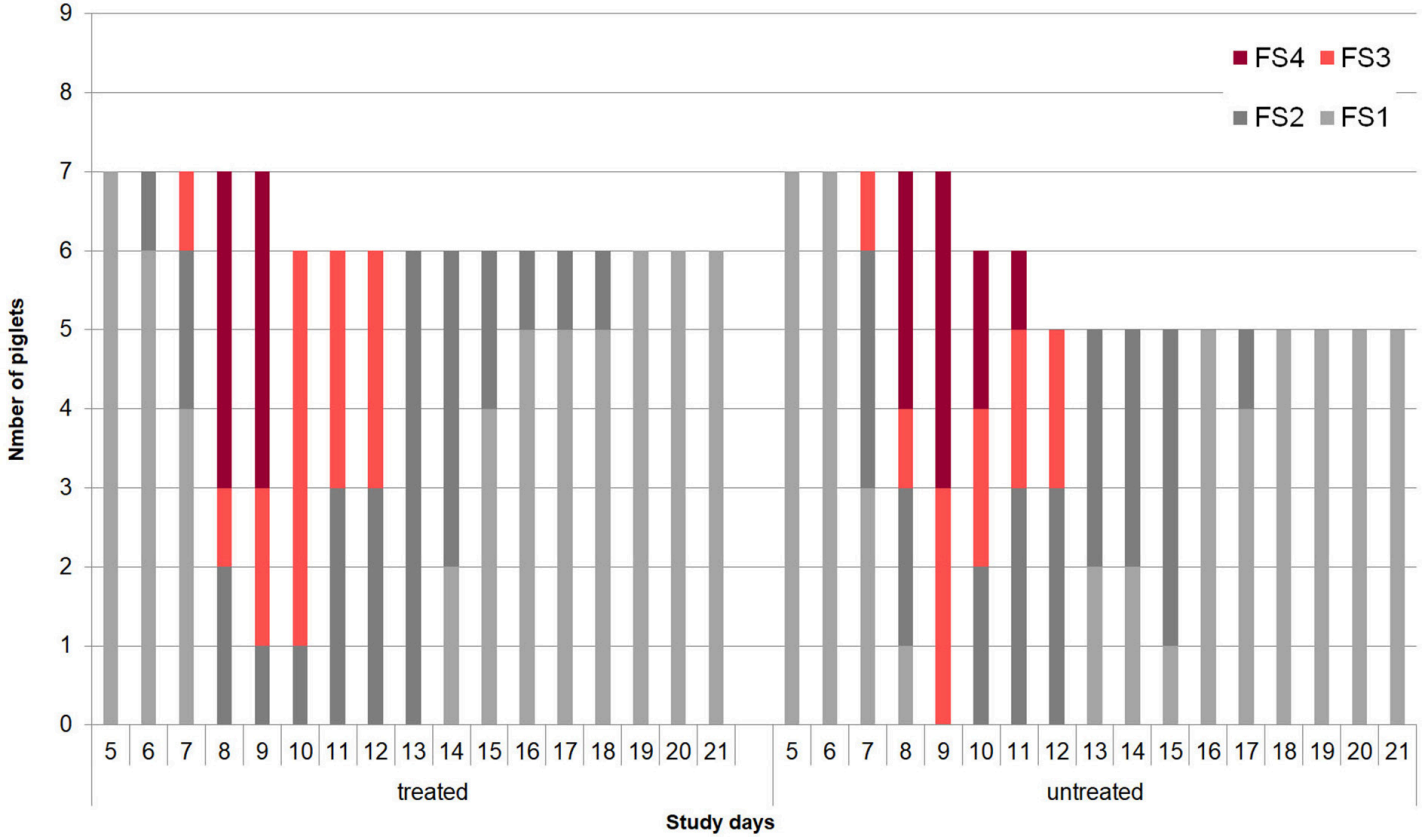
Development of individual fecal scores (1 = firm, 2 = pasty, 3 = semi-liquid, 4 = liquid) in the treated (*n* = 9) and untreated (*n* = 9) piglets in Trial 2.

**Figure 3:**
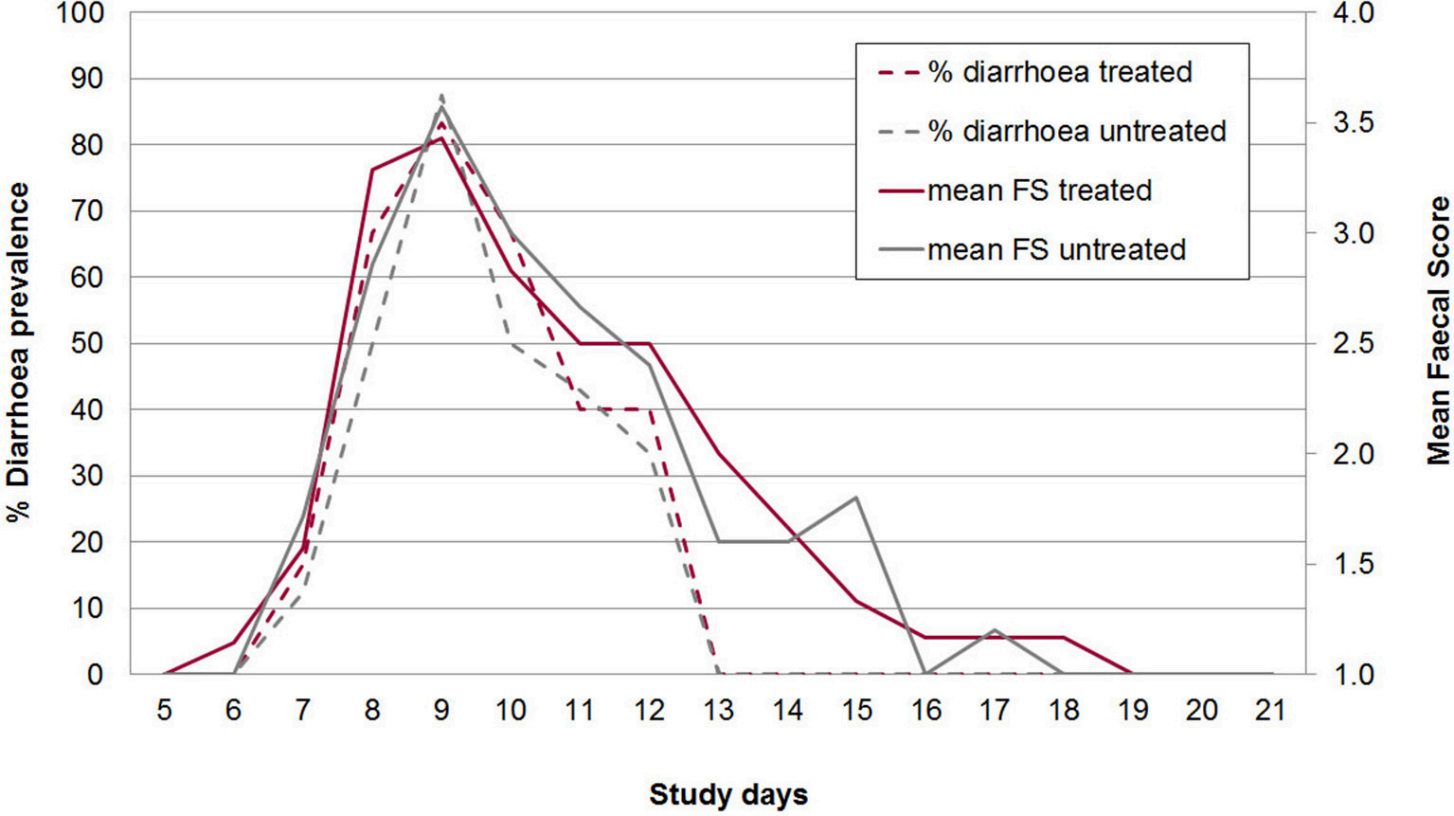
Mean diarrhea prevalence (broken lines) and mean fecal score (1-4 with 3 and 4 = diarrhea; full lines) from study days 5–21 in treated and untreated, *C. suis*-infected piglets (Trial 2). Piglets were treated/sham-treated as described for Trial 1 and infected on the same day with 1,000 sporulated oocysts of *C. suis*.

No adverse effects on piglet health due to oral application of the bacterial cocktail directly after birth were observed. At the beginning of the individual daily fecal sampling (SD 5) all piglets has FS = 1. The first piglet showing pasty feces (FS 2) was observed in the control group on SD 6 (Figure 2). The acute phase of *C. suis* infection was characterized by diarrhea which was first detected on SD 7 and peaked on SD 9 in both groups and lasted until SD 12 (Figure 3). No significant differences with respect to FS were examined between treated and untreated piglets.

**Figure 4:**
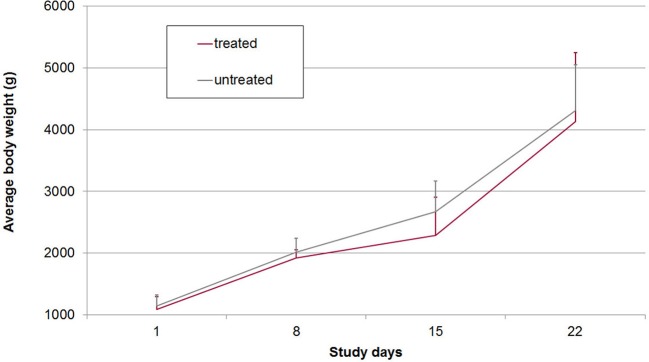
Mean and standard deviation of body weights in treated and untreated, *C. suis*-infected piglets (Trial 2). Piglets were treated/sham-treated as described for Trial 1 and infected on the same day with 1,000 sporulated oocysts of *C. suis*.

On average 20.0% of the samples in group A2 (*n* = 90) and 22.8% of the samples in group B2 (*n* = 114) had FS 3 or 4 (diarrhea). All piglets had diarrhea for at least one day (mean diarrhea days in group A2: 3.0, in group B2: 3.3). Daily body weight gain was reduced around the peak time of diarrhea but increased again in the 3rd week of life (Figure 4). There were no statistically significant differences in mean body weights between both groups on SD 1, SD 8, SD 15, and SD 22, respectively (Figure 4). The average daily weight gain of the piglets from SD 1 to SD 22 was 145 g (standard deviation: 106 g), in group A2 and 150 g (standard deviation: 179 g) in group B2 without statistical significance (*p* > 0.05).

### Mortality

Four piglets in trial 2 (2 out of group A2 and 2 out of group B2) were crushed by the mother on SD 2. Two further piglets (one from each group) were found dead on SD 9. Both had shown a FS 4 (watery diarrhea) on the two last days. Necrotic enteritis was detected during gross necropsy.

### Oocyst Excretion

**Figure 5:**
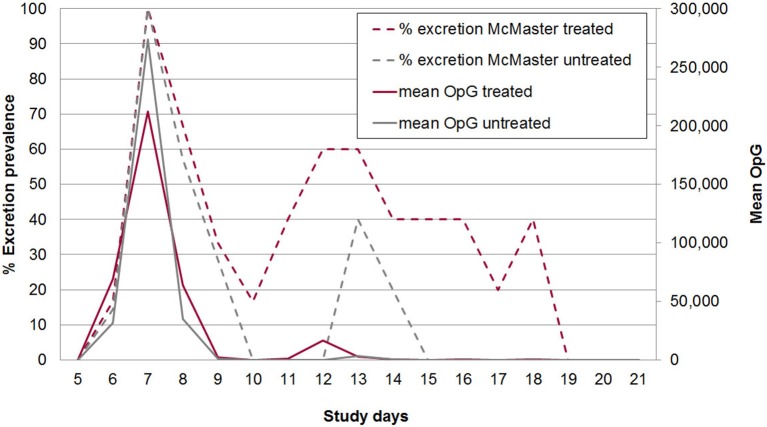
McMaster-countable oocyst excretion prevalence (broken lines) and mean excretion intensity (oocysts per gram of feces; OpG) from SD 5 to 21 in treated and untreated, *C. suis*-infected piglets (Trial 2). Piglets were treated/sham-treated as described for Trial 1 and infected on the same day with 1,000 sporulated oocysts of *C. suis*.

The acute phase of infection was characterized by autofluorescence-detectable oocyst excretion from SD 6 to 18. Master-countable oocysts (individual daily samples) could be found from SD 6 to SD 18 in group A and from SD 6 to SD 14 in group B with a peak in prevalence and mean OpG on SD 7 in both groups (Figure 5). All piglets were positive for excretion for at least one day. In total 51.1% of the samples in group A2 were positive for oocysts in autofluorescence, 35.6% were McMaster countable, while in group B2 38.5% of samples contained oocysts countable in 19.3% of the samples. On average animals excreted oocysts for 7.7 days in group A2 and 5.6 days in group B2. Also the area under the curve for the total OpG was slightly higher in group A2 (369.479, standard error: 202.805) than in group B2 (344.598, standard error: 166.671) (Figure 5).

## Discussion

The beneficial effects of probiotics are deduced from diverse mechanisms such as modulation of host immunity, strengthening of the intestinal barrier by increase of intestinal antimicrobial activity and Paneth cells, competitive exclusion of pathogens by interference with space and nutrients or production of antimicrobials (32, 33). Due to increasing efforts to reduce antibiotic use in pig production in order to minimize risk of development of bacterial resistance against antimicrobials, application of beneficial microorganisms for alternative treatment of piglet diarrhea received increasing attention (33).

In the present study the effect of pig-derived probiotic bacteria on the health of newborn piglets was investigated. In the absence of other enteropathogens, application of probiotics improved weight gain indicating a beneficial effect. In the presence of the protozoan pathogen *C. suis*, however, this effect was not detectable, and both probiotic-treated and untreated piglets developed signs of clinical cystoisosporosis with semi-liquid to liquid, non-hemorrhagic diarrhea.

In the first trial, the overall vitality of the piglets of two litters was reduced by congenital defects (splay legs) and may have interfered with the beneficial effect of the probiotic cocktail; in these litters no positive treatment effects could be observed. This reflects that negative effects of lower birth weight and vitality can superimpose any potential positive treatment effects. Especially splay legs are a multifactorial disease due to genetic, developmental and environmental factors decreasing vitality in piglets. In addition, other parameters such as colostrum uptake and quality influence the development of microbiota in piglets by itself and is again highly dependent on birth weight and vitality (34–36). Under practical conditions probiotic treatment can therefore not compensate for any other health and management problems present on farm.

In the second trial 4 out of 18 piglets were crushed until SD 2. This mortality rate of 22% is comparable to mortality observed in the first week of suckling on farms without specific disease problems. Maternal behavior and suboptimal flooring systems are the cause for crushing without accompanying disease on conventional farms. The two piglets that died on SD9 belonged to both groups and showed necrotic enteritis typical of severe cases of cystoisosporosis (12).

*Cp*A as the first colonizer of the piglet gut rapidly decreases in numbers under physiological conditions until weaning (3). Inoue et al. observed a decrease of *Cp*A aas early as day 4 of life (4). This decrease might be due to the parallel increase of maternal immunoglobulin A as a major component of lactogenic immunity (37). In the second and third week of life the diversity of anaerobic bacteria increased (35). After the third week of life the shift from aerobic and facultative anaerobic toward an anaerobic flora is nearly completed, accompanied by a marked decrease in *E. coli* and further maturation of the gut (4, 37, 38). In case of infections with *C. suis* in the first days of life, the developmental succession of the piglet gut ecosystem might be disrupted, because destruction of epithelial cells during the intraepithelial multiplication of the parasite can predispose for colonization of alternative microorganisms. In chicken, infection led to increased mucus production, which favors growth of *C. perfringens* (22). Until now, the question whether the order of infection of the two pathogens—*C. suis* and *Cp*A—is decisive for the outcome of disease or whether virulent strains of *Cp*A exist which predispose for severe forms of coccidiosis has not been answered. Since *Cp*A belongs to the physiological gut flora in the first days of a piglet's life, the hypothesis of the study was that application of *Cp*A strains prior to infection with *C. suis* might prevent a severe course of neonatal cystoisosporosis. To exclude an adverse effect of the artificial bacterial cocktail, its effect on piglet development and well-being was evaluated in the first trial. Although only four litters were included in this trial, a significant positive effect on growth rate could be confirmed in the absence of notable negative effects on piglet health. In the second trial we evaluated clinical and parasitological parameters in two groups of suckling piglets infected with *C. suis*, one treated with a probiotic cocktail and a non-treated control group from the first day of life (also the day of infection) until three weeks of life. Piglets did not show adverse reactions after cocktail application. Clinical and parasitological findings of piglets of the control group were similar to published results in previous studies, in which piglets had been also infected in the first four days of life with 1,000 oocysts of the same strain used in this study (39–41). While clinical parameters in piglets treated with the probiotic cocktail and control piglets did not notably differ, there were obvious, although statistically not significant, differences in oocyst shedding. Piglets treated with probiotics showed prolonged oocyst shedding (two days longer on average) and a higher prevalence of shedding piglets during the second peak of infection, while the estimated total excretion (AUC of OpG) was similar in both groups and the maximum OpG was higher in the treated group. Therefore, no impact of non-pathogenic *Cp*A and *E. coli* strains on the course of infection with *C. suis* could be deduced from the results of the trial—neither for improvement nor for aggravation of the clinical outcome of cystoisosporosis, and the underlying hypothesis of a beneficial effect of an early competitive colonization with probiotic bacterial strains prior to contact with pathogens had to be rejected for this model. Although *Clostridium* spp. belong to the physiological gut flora of suckling piglets and have anti-inflammatory properties within the gut microbial community (5), any potential positive effect might have been superimposed by the pathogenic effects of infection with *C. suis*. In general, the health-promoting effects of probiotic bacteria depend on neutralization of effects of pathogens by various mechanisms, as blocking of colonization sites, reinforcement of tight-junctions, mucus induction, and stimulation of innate immune responses (42, 43). The intestinal development of *C. suis* starts with excystation of sporozoites in the gut lumen and their intrusion into enterocytes (44). It can be assumed that neither attachment mechanisms of probiotic *E. coli* nor bacterial adherence of interfered with the specific infection mechanisms of *C. suis*. Subsequent multiplication takes place inside the host cells, in a different compartment from the contemporaneous development of the microbial ecosystem of the gut. In the case of *Cp*A replication is restricted mainly to the gut lumen without a tight association to the epithelium, and toxin production (rather than tissue invasion) is known to be decisive for development of disease (45). It is therefore reasonable to assume that the parasite remains mostly unaffected by microbiota within its ecological niche as shown in previous experiments with gnotobiotic vs. conventionalized piglets (46, 47). However, it is puzzling that even under experimental conditions *C. suis* infections do not induce disease in all piglets alike (24) and that not only the amount of oocysts shed but also the level of diarrhea is strongly dependent on the age of the piglets at the time of infection (30, 39).

It has been hypothesized that this phenomenon is correlated with the disruption of the development of the physiological gut flora and the colonization of the parasite gut with enteropathogens (39). In a case-control study on diarrhea in piglets *C. suis* but not *C. perfringens* was significantly correlated with diarrhea, and a significant increase of diarrhea was noted in the presence of *C. suis* and beta2-toxigenic *C. perfringens* (15). Recently, interactions between *Cp*A and *C. suis* have been described in experimental infections with *C. suis* and result in an exacerbation of clinical signs in affected piglets, especially in early life (25). So far it is unclear whether this influence is direct or indirect, mutual or one-sided and, if the latter is the case, whether the parasites or the bacteria are the drivers. Application of early anticoccidial treatment relieved the symptoms of diarrhea and reduced the tissue adherence of *Cp*A (25); however, the exact mechanisms of this effect are unclear. In the present study we applied non-pathogenic bacteria simultaneously with *C. suis* infection which had no pronounced influence on the clinical course of coccidiosis including oocyst shedding. A possible influence of parasite infection on disease development in pigs colonized by *Cp*A needs to be further investigated in a larger number of animals. In chicken, necrotic enteritis due to *C. perfringens* can be controlled by proper coccidiosis management (22), and previous works indicated that chemotherapeutic control of piglet cystoisosporosis can reduce the use of antibiotics for the treatment of enteral diseases in piglets (48).

In contrast to *Cp*A, the role of enteropathogenic *E. coli* in the exacerbation of piglet coccidiosis has not yet been confirmed experimentally. Enterotoxic *E. coli* are not regularly present during coccidiosis (49–51), and in very young piglets seems to be absent in the presence of clostridia (13). It must be assumed that *E. coli* is not strongly affected by *C. suis* infections or vice versa.

## Conclusion and Outlook

Application of the used bacterial probiotic cocktail improved the growth of healthy piglets while it had no influence on the course of experimental *C. suis* infections in neonates. More research is needed to determine the exact mechanisms of pathogen displacement by probiotics and of bacteria-parasite-host interactions in the early phase of life to be able to efficiently control neonatal diarrhea as a multifactorial disease.

## Author Contributions

CU: Sampling and analysis of piglets in Trial 2, drafting of the manuscript; LS and MV: Sampling and analysis of piglets in Trial 2; AvA: Sampling and analysis of piglets in Trial 1; GG: Provision of the cocktail material for both Trials; K-HW: Planned and supervised Trial 1; AJ and IH-P: Designed the experiments and revised the manuscript drafts. All authors approved of the final version of the submitted manuscript.

### Conflict of interest statement

The authors declare that the research was conducted in the absence of any commercial or financial relationships that could be construed as a potential conflict of interest.
